# Toward a Comprehensive Pea Aphid Saliva-Proteomewith Insights from Transcripts from the Whitefly *Bemisia tabaci*

**Published:** 2020-04-13

**Authors:** Gerald Reeck, Raman Chandrasekar, Matthew Aksamit, Doina Caragea, Xiao-Wei Wang, Shu-Sheng Liu, Le Kang, Feng Cui, James Balthazor

**Affiliations:** 1 Department of Biochemistry and Molecular Biophysics, Kansas State University, USA; 2 Department of Computer Science and Information, Kansas State University, Manhattan KS 66506 USA; 3 Institute of Insect Sciences, Zhejiang University, Hangzhou 310058, China; 4 State Key Laboratory of Integrated Management of Pest Insects and Rodents, Institute of Zoology, Chinese Academy of Sciences, Beijing 100101, China; 5 Department of Chemistry, Fort Hays State University, Hays KS 67601 USA

**Keywords:** Pea Aphid, Saliva, Proteins, Transcripts, Genes, Saliva-Proteome, Transcriptomics, Proteomics, Whitefly

## Abstract

The aim of this research project is to compile and analyze numerous sources of transcriptomic and proteomic published works into an initial attempt at a comprehensive salivary transcriptome with considerations into various insect species. Published transcriptomic and proteomic results with RNA-Seq analysis of pea aphid salivary gland RNAs were used to create a comprehensive catalog of pea aphid *(Acyrthosiphon pisum)* salivary proteins. Duplications of transcripts within the approximately 15 referenced transcriptomics-based papers were eliminated, as were duplications within the approximately 10 referenced proteomics-based papers and duplications between transcriptomic and proteomic work. For studies that had been done in aphids other than the pea aphid, orthologs in the pea aphid were identified by blast searches. The result is a proposed 131-component saliva-proteome for the pea aphid, 45 entries of which were identified by proteomics and 102 by transcriptomics, with 16 proteins (and their encoding transcripts) having been identified in both approaches. Transcripts encoding nearly all of the proposed saliva-proteins were verified in pea aphid salivary glands by RNA-Seq analysis. In light of the complexity of the proposed saliva-proteome, we suggest that many different salivas can in fact be produced by a pea aphid by combining different components of the saliva-proteome. Each salivation would be a subset of the total saliva-proteome, customized to meet a particular physiological condition that the species encounters. Thus, the proposed saliva-proteome can provide a basis for systems-level studies of aphid salivations. Finally, we to show orthology into other insect species, it was found that approximately 80 components of the proposed pea aphid saliva-proteome have orthologs in a whitefly, *Bemisia tabaci*. This suggests that a common core of orthologous proteins and enzymes exists in the salivations of aphids and whiteflies.

## Introduction

It has long been recognized that saliva plays a central role in aphid feeding on host plants [1]. There has been a particular focus on the proteins of saliva, since researchers have shown that salivary proteins play a key role as key actors on the aphid side in aphid/plant interactions [2,3]. Just as saliva proteins may enable feeding on a host plant, a failure of salivary proteins to maintain feeding or to overcome plant defenses may well limit feeding on a non-host plant species (or cultivar). In this view, achieving a detailed understanding of the action of individual proteins and enzymes of aphid saliva in host and non-host plants will ultimately make a major contribution to our understanding of aphid/plant co-evolution and to the development of new control strategies for species that feed on crop plants.

The proposed research will develop a comprehensive inventory of saliva proteins and their encoding transcripts and genes. This list is termed a saliva-proteome. More specifically, our goal is to reliably and completely identify the saliva-proteome for a given aphid species, namely the pea aphid *(Acyrthosiphon pisum)*, selected because this species remains the most advanced genetic model among aphids, a published genome sequence [4]and with many ESTs, sequenced in numerous laboratories and collected and annotated at AphidBase (www.aphidbase.com). The current research will serve as a template for saliva-proteomes of other aphids and, potentially, closely related Hemipteran. Identifying a complete saliva-proteome, even for an individual species, is a long-term goal with many steps along the way, and this paper is intended to be one of those steps.

Several methods have been used in identifying proteins of aphid saliva. The first approach, described by Miles et al. [3], was based on assays of enzyme activities in saliva. As important as that line of investigation was, rather limited conclusions ultimately were drawn from it, and understandably so. The enzyme assays were typically carried out on unfractionated and highly diluted saliva collected from the feeding and salivation of aphids on artificial liquid diets. If a particular enzymatic activity was in fact detected (and detection itself was not necessarily easy, given the dilute nature of the samples), the result could not be interpreted at the level of individual gene products. That is, several individual gene products could in principle contribute to any given enzymatic activity. A further and severe limitation of enzyme-based studies is that by their very nature they could not hope to identify saliva proteins that are not enzymes. As we shall see, this is a major limitation.

In this millennium, numerous laboratories have turned to more powerful approaches, particularly transcriptomics [5–7] and proteomics [8–10]. These approaches allow identifications of enzymatic and non-enzymatic proteins at the gene level. Protein c002, for instance, is a prototypical non-enzyme saliva-protein, and it has been shown to be required for feeding on a host plant [11–13].

Here a combination results from numerous studies results obtained by several methods and on several aphid species by numerous research groups. Our underlying hypothesis is that there may be many commonalities in the saliva-proteomes of various aphid species. Once having proposed a pea aphid saliva-proteome, we establish by RNA-Seq analysis the existence of nearly all of the encoding transcripts in pea aphid salivary glands, regardless of the aphid species in which the original transcript or protein was identified. The result is a proposed pea-aphid saliva-proteome of 131 proteins (and their transcripts and genes). Given this considerable complexity, we suggest that many different salivas can be secreted by the pea aphid by synthesizing sub-sets of its saliva-proteome.

Whiteflies, including *Bemisia tabaci*, are piercing/sucking insects that are grouped along with aphids and psyllids in the suborder Sternorrhyncha. Numerous whiteflies are major crop pests and are thus under intensive investigation. As close relatives of aphids, whiteflies are particularly appropriate for comparisons with aphids at the molecular level, looking for correspondences (that is, orthologies) and, as well, lack of correspondences. Such comparisons could help identify similarities and differences in mechanisms of feeding on host plants by aphids and whiteflies. As a starting point, we here compare the predicted proteins of our proposed pea aphid saliva-proteome with proteins predicted from *Bemisia tabaci* transcripts, and find a strikingly large set of correspondences (putative orthologies) along with interesting non-correspondences (apparent lack of orthologs).

## Methods

Gathering data from transcriptomics studies, our starting point in assembling a list from transcriptomics studies was our earlier list of pea aphid ESTs as presented in Carolan et al., and in particular, the list of 42 transcripts of in Table 1 of that paper. We chose this as a starting point because of the requirement, for inclusion in that list, not only of a predicted signal secretion peptide, but also of enrichment in libraries constructed from RNA from pea aphid salivary glands with respect to whole-body RNA preparations. Forty-one of those transcripts are entered in Table 1 of this paper (and are coded T1) and thus serve as the foundation for the “transcriptomics-based master list” of this paper. (We note that one entry in Table 1 of Carolan et al. no longer exists as an AphidBase entry and it is therefore not included in Table 1 of this paper.)

In Carolan et al. we evaluated salivary-gland enrichment using the R-statistic of Stekel et al. [14]. In this paper, as an initial step in expanding the list of transcripts believed to encode saliva-proteins, we relaxed our criterion for salivary-gland enrichment by lowering the cut-off R value from 7 to 5. This relaxation is consistent with our overall approach here of preferring to include “too many” rather than “too few” transcripts (and their encoded proteins). This step added 8 transcripts (coded T2) to our “transcriptomics-based master list.”

Additional entries to the master list at the transcriptomics level came from Ramsey et al., Bos et al., Cui et al. and Atamian et al. [5–7,15]. These entries are coded T3 through T6 in Table 1 of this paper. As we proceeded through those papers, duplications from earlier papers were eliminated.

To be included in Table 1, an encoded protein must be predicted to contain an N-terminal secretion and to lack a membrane anchor sequence, both as predicted by Signal P (http://www.cbs.dtu.dk/services/SignalP-3.0). We have intentionally used a rather weak cutoff, namely a probability of 0.5 or higher, for predicted secretion signals. This is in the spirit of our overall approach, which, as indicated above, is to err on the side of possibly including too many (rather than too few) transcripts and their encoded proteins. At the same time, we note that the vast majority of the encoded proteins have secretion-signal prediction probabilities of 0.85 or greater. Another relevant issue is the occurrence of endoplasmic reticulum retention signals. We have retained in the master list a few proteins with apparent ER retention signals. This inclusion is based in large part on our having found that the protein Armet has an ER retention signal but was also found to be a protein of pea aphid saliva [16]. In other words, it appears that some ER retention signals are “leaky,” and that a secreted protein with such a signal can function extracellularly.

In papers on aphids other than the pea aphid and in which the authors did not identify pea aphid orthologs, we identified the pea aphid orthologs (by searching with BLASTn against pea aphid nucleotide sequences) for inclusion in our table.

### Gathering data from proteomics studies.

Proteins identified in aphid saliva by proteomics come from Harmel et al., Carolan et al., Cooper et al., Rao et al., and Vandermoten et al. [8–10,13]. To enter a protein into our “proteomics-based master list” (Table 2), we again applied the criteria that a protein (whose entire sequence was obtained by translation of its encoding mRNA) must have a predicted N-terminal secretion signal and must lack a predicted N-terminal membrane anchor sequence. Because their work was in the pea aphid, we began construction of this master list with the proteomics paper of Carolan et al. Proteins (and their encoding transcripts) from that paper are coded as P1 in our Table 2. The other entries in that table are taken from the remaining proteomics papers in chronological order. We eliminated duplications as we proceeded chronologically through the papers. We note here that, of the numerous proteins identified by Rao et al. in saliva of three cereal aphids, pea aphid orthologs had been previously identified in other proteomics studies and therefore there are no entries in Table 2 from Rao et al. For studies in species other than the pea aphid, pea aphid orthologs were identified using BLASTn.

### RNA-Seq of pea aphid salivary glands.

Aphids for these studies were maintained on fava beans. Diet-fed aphids were transferred to Akey-Beckdiet for 24 h, RNA was isolated from over 400 pea aphid salivary glands (half from plant-fed and half from diet-fed insects) that had been dissected into RNAlater (Life Technologies). For RNA isolation we used the QIAzol reagent (Qiagen, Valencia CA), along with the Qiagen RNAeasy Kit for eliminating contaminating DNA. The quality of the purified RNA was examined using the Agilent Bioanalyzer 2100. Further processing of the samples was done in the Integrated Genomics Facility at Kansas State University. cDNA libraries were constructed using Master Mix kits of Life Technologies. Reads, of 250 bases, were obtained on the Illlumina MiSeq Platform. RPKM values are obtained from 140 million reads. Assembly of reads was performed with Geneious software, using default settings and, as templates, the “gene set” for the pea aphid (that is, all pea aphid transcripts from AphidBase).

### Comparison with Bemisia tabaci transcripts.

We conducted tBLASTx searches at NCBI using each of the 131 transcripts of our proposed pea aphid saliva-proteome as a query sequence and limiting the search (within the “nr” database) to *Bemisia tabaci*. A hit from a tBLASTx search is taken as a putative ortholog if it was a top hit in the search and had an e-value of 10^−15^ or less. Annotations for such sequences are taken from NCBI.

## Results

### Creating a transcriptomics-based “master list”.

Using the approach described in Methods, we created a non-redundant list of proteins that have been proposed to be components of aphid saliva based on transcriptomics approaches. The starting point for the transcriptomics-based portion of the saliva-proteome was the list of 42 transcripts that we previously identified as significantly enriched in pea aphid salivary gland cDNA libraries compared to whole-body cDNA libraries (Table 1). This list was expanded as described in Methods. When authors did not identify pea aphid orthologs, we did so using tBLASTn searches against pea aphid nucleotide sequences at NCBI. Using all available sources, we accumulated 102 non-redundant pea aphid transcripts that had either been proposed as pea aphid saliva components or are orthologs of transcripts proposed as saliva components in other aphid species (Table 1).

In Table 1, we indicate: the ACYPI transcript identification number; a coding for the source-paper for each entry; the pea aphid gene number from NCBI; the description of the transcript as given in the source-paper; and the annotation at AphidBase/NCBI.

### Creating a proteomics-based “master list”.

Next we created a separate list, working from proteomics studies typically conducted on saliva, but in one case with extracts of salivary glands. We took as our first source (coded as P1 in Table 2) the work of Carolan et al. [17], because their work was with saliva from the pea aphid itself. For each entry we imposed the same criteria as with the entries in Table 1, namely that the encoding transcripts encoded a predicted N-terminal secretion signal and did not encode an N-terminal membrane anchor.

We built the proteomics-based master list in steps, paper by paper, adding the results of Cooper et al., Carolan et al., Rao et al. and Vandermoten [8,10,13]. The result was a non-redundant list of 45 saliva proteins (Table 2) identified by proteomics.

### The proposed saliva-proteome and RNASeq analysis.

To obtain our proposed pea aphid saliva-proteome, we combined the transcriptomics-based master list and the proteomics-based master list. The resulting list is shown in Table 3. A total of 16 transcripts/proteins had occurred in both the transcriptomics-based master list and the proteomics-based master list. These carry both T and P designations in Table 3, which comprises 131 proteins and their encoding transcripts and genes. In this Table 3 we indicate the predicted secretion-signal probabilities, along with current transcript and gene identifications, ER-retention signals for several proteins, literature-sources and annotations drawn either from NCBI or Aphid Base.

Some of the entries in Table 3 came from studies of aphids other than the pea aphid. Orthologs were identified for such proteins among pea aphid entries at Aphid Base. In other words, we know that the transcripts in Table 3 occur in the pea aphid. But the question remains: Do these transcripts occur in *salivary glands* in the pea aphid? To address this question, we conducted RNA-Seq analysis of RNA isolated from salivary glands dissected from pea aphids. As shown in Table 3, based on RPKM values, we can see that nearly all the listed transcripts are in fact present in pea aphid salivary glands, although as might be expected, the RPKM values for the various transcripts vary widely.

### Pea aphid/whitefly comparisons.

Table 4 lists orthologs we have identified between components of the pea aphid saliva-proteome and proteins encoded by transcripts from *Bemisia tabaci*. These correspondences were discovered by tBLASTx searches as described in Methods. Of the 131 predicted proteins of Table 3, many (78 or nearly 60%) have orthologs among *Bemisia* transcripts in that they encode proteins with strong sequence similarity to proteins encoded by *Bemisia* transcripts. In Supplemental Table 1, we show alignments of several *Bemisia* proteins with components of the pea aphid saliva-proteome.

## Discussion

The immediate impression from the proposed pea aphid saliva-proteome of Table 3 is its complexity. It appears that the pea aphid has many and diverse proteins and enzymes to use in forming saliva. This leads us to reconsider the nature of aphid saliva.

Instead of thinking of an aphid as being able to make one saliva or, in a somewhat more sophisticated view, two salivas (sometimes called “watery” and “gelling“ salivas), we propose that an aphid species can produce *many* salivas. Each saliva would differ in its makeup that is, in the proteins and enzymes that it contains, or, more generally, in the proportions of its components.

Different salivas may well be needed by an aphid under the range of conditions it faces in its lifetime. These could include:

- Different stages or phases of feeding on a host plant (and perhaps different sub-phases as represented by different wave-forms in electrical penetration graph studies [18]
- Feeding on different host plants [19,20]
- Attempted feeding on various *non-host* plants
- Feeding on artificial diets, including various artificial diets of different compositions and therefore of different abilities to support an aphid species’ growth and reproduction

As regards differences from one aphid species to another, we must keep in mind that orthologs in two species could differ subtly in function. Thus, a particular protein (say, Protein c002) is reasonably called by the same name in different species but is not, of course, identical in different aphids [2,7]. Thus, two salivas of the same composition in two species can be presumed to be uniquely adapted, through the evolution of the amino acid sequences of its components, to establish the evolutionary fitness of each species.

Turning to more technical matters, our proposed saliva-proteome in Table 3 contains 131 components, each of which has been suggested as a protein of saliva (often through studies of its encoding transcript) by at least one research group and, in numerous instances, by two or more research groups and in 42 cases by both transcriptomic and proteomic methods. Of course, there are likely errors of both omission and of inappropriate inclusion in our proposed saliva-proteome. As regards the latter, proteins of extracellular-matrix proteins could satisfy our criteria for inclusion in Table 3, and, unless such a protein served a dual role, it will ultimately be excluded from the saliva-proteome. Thus, Table 3 should be considered as a working proposal, for the community of aphidologists to refine in ongoing work.

Our RNASeq analysis provided evidence in the large majority of cases for the existence of the transcripts in Table 3 in the *salivary glands* of the pea aphid. In cases where the RPKM value is particularly low, it could be that the saliva of the aphids feeding on fava beans did not require the protein encoded by such transcripts. In refining our proposed saliva-proteome, we will need to apply different criteria not only to cases of low RPKM, but more broadly throughout the 131 components. In other words, the list in Table 3 is a “state of the art” view, and it will no doubt be refined in future investigations, probably removing some proteins in Table 3 and possibly adding others to that list.

Finally among the technical points, we would like to add a note of caution or realism. In very few cases – and in the case of no enzyme, to our knowledge – have the proteins listed in Table 3 been expressed and studied at the protein level. The single most thoroughly studied component at the protein level is Armet [21]. In that case, studies of the protein itself (and not just similarity in sequence of a predicted protein to its mammalian orthologs) allows us to call this protein Armet in the pea aphid. Similarly, to our knowledge, only in the case of Armet has the location of the cleavage of a signal peptide been shown directly. The other entry that has been studied at the protein level is the cysteine-rich protein encoded by transcript ACYPI139568, which Guo et al. demonstrated by direct measurement to be a zinc-binding protein. In all other cases, the names in Table 3 should be seen to implicitly include the qualifying term “like” even when the term is not explicitly included in the annotation.

The availability of transcriptomic data in the whitefly *Bemisia tabaci* has allowed us to ask an important question: Of the 131 components of the proposed pea aphid saliva-proteome, how many can be said to have orthologs in Bemisia? This begins to answer the question of the extent of correspondence between saliva-proteomes of these two species, both from the sub-order Sternorrhyncha, but from different superfamilies (Aleyrodoidea and Aphidoidea). We see this as a starting point for a broader study of correspondences (and the lack of correspondences) in the saliva-proteomes throughout the order Hemiptera.

The results of tBLASTx searches, presented in Table 4 strongly suggest that there is a common core of orthologous enzymes and other proteins in the saliva-proteomes of the pea aphid and *Bemisia tabaci*. It is also the case that numerous proteins in Table 3 do not appear to have orthologs in Bemisia. For instance, Protein c002, cysteine-rich protein, and several other “unknown proteins” of Table 3 had no matches among translated Bemisia transcripts. But Table 4 suggests that we might think of the two species as having a common core to their saliva-proteomes, much as has been suggested by Thorpe et al. for 3 aphid species [22].

Among the pea aphid and Bemisia orthologs for which we show alignments (see Supplemental Material), we would like to discuss two here. Jiu et al. characterized two glutathione peroxidase transcripts in *Besimia tabaci* [23]. One (BtPHGPX-1) is matched in Table 4 to pea aphid glutathione peroxidase-1 (and the two amino acid sequences can be seen in alignment in the paper by Jiu and coworkers). Since the publication of that article, NBCI, in re-annotating the pea aphid genome, identified a second putative glutathione peroxidase, called it glutathione peroxidase-2. This predicted enzyme is matched with BtPHBPX-2 in Table 4. Thus, there are two pairs of putatively orthologous glutathione peroxidases from the two species. Alignment of the glutathione peroxidase-2 and BtPHGPX-2 is given in Supplemental Material. How the glutathione peroxidases-1 and glutathione peroxidases-2 might differ within a species for instance in substrate specificity remains to be established. All four enzymes have predicted N-terminal secretion signals, so it seems likely that all are components of saliva.

As detailed by Rao et al. glucose dehydrogenase has been reported frequently as a component of aphid saliva. The same authors suggest that glucose dehydrogenase might be involved in lowering the concentrations of reactive oxygen species produced as a part of plant defense. Table 4 contains several straightforward matches of glucose dehydrogenases from the pea aphid and Bemisia, but there is one particularly interesting and less obvious match. Pea aphid transcript XM_001949497.3 is annotated at NCBI as uncharacterized (“unknown,” in our terminology). In our tBLASTx searching, a strong match occurred between this entry and Bemisia transcript XM_019055567.1, which is annotated as glucose dehydrogenase. The two predicted proteins are quite different in length (XXX amino acid residues for the pea aphid protein and YYY for the Bemisia protein). The latter is a typical length for a glucose dehydrogenase. Alignment of the amino acid sequences (see Supplemental Material) is very strong but is limited to the C-terminal half of the pea aphid protein. Broader BLAST searches (results not shown) indicated that proteins with a glucose-dehydrogenase C-terminal half and a predicted length of the pea aphid protein are restricted to aphids. The results suggest that at least some aphids have a form of glucose dehydrogenase with a several hundred residue N-terminal region of unknown function. One possibility is that this N-terminal region serves as a binding domain that could serve to localize the enzyme, presumably within sieve elements.

Comparative work, whether it is between an aphid and a whitefly or between different aphid species needs to be qualified in the following sense: orthology is not identity. The “common” elements of aphid saliva as suggested by Thorpe et al. in their interesting paper on the comparison of three aphid species (not including the pea aphid) or the commonalities suggested here between the pea aphid and Bemisia are orthologies. That means a common ancestral origin of 2 genes (and their encoded transcripts and proteins). But orthologous proteins are virtually never identical proteins and even in such a case the underlying genes and transcripts will not be identical. In other words, orthologies in fact embody differences. Faced with similarities rather than identities, the default assumption should be that each genetic triad (gene/transcript/protein) is adapted to a function and to control that are, in detail, unique to its species. Thus, one could imagine that two species could have saliva-proteomes with perfect one-to-one correspondence (each genetic triad in each species being orthologous to just one triad in the other species) but that each such saliva-proteome (with its underlying transcripts and genes) would be uniquely adapted to its species. As we extend our comparisons, among aphids and other hemipterans, species-specific adaptations of orthologs should be kept in mind [24,25].

## Conclusion

Finally, we note the recent paper by Zhang et al. which reports over 33,000 unigenes from their transcriptomic analysis of the salivary glands of the grain aphid, over 500 of which are predicted to encode proteins of saliva. We have these results under investigation at the current time.

## Supplementary Material

alignments

## Figures and Tables

**Table 1:** Transcriptomics-based Master List

	Gene ID	Transcript ID	Code	Annotation in Source	Annotation in AphidBase
1	100158867	ACYPI000288	T1	glucose dehydrogenase	glucose dehydrogenase [FAD, quinone]-like
2	100159063	ACYPI000472	T1	unknown protein	uncharacterized LOC100159063
3	100159087	ACYPI000490	T1	unknown protein	uncharacterized LOC100159087
4	100159160	ACYPI000558	T1	unknown protein	putative uncharacterized protein DDB_G0282133
5	100575164	ACYPI000733	T1	dipeptidyl carboxypeptidase	angiotensin-converting enzyme-like
6	100159485	ACYPI000852	T1	unknown protein	uncharacterized LOC100159485
7	100159632	ACYPI000986	T1	glucose dehydrogenase	uncharacterized LOC100159632
8	100159750	ACYPI001099	T1	unknown protein	uncharacterized LOC100159750
9	100159805	ACYPI001152	T1	unknown protein	uncharacterized LOC100159805
10	100159932	ACYPI001271	T1	unknown protein	uncharacterized LOC100159932
11	100160226	ACYPI001541	T1	unknown protein	uncharacterized LOC100160226
12	100160301	ACYPI001606	T1	unknown protein	uncharacterized LOC100160301
13	100160421	ACYPI001719	T1	unknown protein	uncharacterized LOC100160421
14	100160554	ACYPI001843	T1	unknown protein	probable serine/threonine-protein kinase clkA
15	100160601	ACYPI001887	T1	unknown protein	uncharacterized LOC100160601
16	100160906	ACYPI002172	T1	unknown protein	trichohyalin-like
17	100161198	ACYPI002439	T1	glutathione peroxidase-1 (Me23)	uncharacterized LOC100161198
18	100161239	ACYPI002476	T1	inositol monophosphatase	3′(2′),5′-bisphosphate nucleotidase 1-like
19	100161690	ACYPI002891	T1	cadherin	protocadherin Fat 4-like
20	100162450	ACYPI003601	T1	unknown protein	micronuclear linker histone polyprotein-like
21	100162547	ACYPI003695	T1	unknown protein	uncharacterized LOC100162547
22	100162791	ACYPI003917	T1	SCP GAPR-1	putative uncharacterized protein DDB_G0277255
23	100163088	ACYPI004198	T1	lipophorin precursor	similar to apolipophorin
24	100164831	ACYPI005818	T1	unknown protein	uncharacterized LOC100164831
25	100165393	ACYPI006346	T1	unknown protein	uncharacterized LOC100165393
26	100166545	ACYPI007406	T1	unknown protein	uncharacterized LOC100166545
27	100166702	ACYPI007553	T1	unknown protein	uncharacterized LOC100166702
28	100167188	ACYPI008001	T1	Armet	mesencephalic astrocyte-derived neurotrophic factor homolog
29	100167427	ACYPI008224	T1	unknown protein (Me10)	uncharacterized LOC100167427
30	100167863	ACYPI008617	T1	Protein c002	uncharacterized LOC100167863
31	100167919	ACYPI008667	T1	unknown protein	uncharacterized LOC100167919
32	100169243	ACYPI009881	T1	putative sheath protein	uncharacterized LOC100169243
33	100169287	ACYPI009919	T1	unknown protein	uncharacterized LOC100169287
34	100574100	ACYPI38795	T1	unknown protein	thrombin-like enzyme cerastocytin
35	100569946	ACYPI39568	T1	aphid specific cysteine rich protein	uncharacterized LOC100569946
36	100302326	ACYPI43360	T1	unknown protein	uncharacterized LOC100302326
37	100570716	ACYPI45001	T1	unknown protein	uncharacterized LOC100570716
38	100159644	ACYPI49603	T1	unknown protein	unknown protein
39	100168118	ACYPI55147	T1	unknown protein	nucleolar protein of 40 kDa-like
40	100162584	ACYPI55148	T1	unknown protein	chondroitin sulfate proteoglycan 4
41	100574994	ACYPI56502	T1	unknown protein	uncharacterized LOC100574994
42	100159394	ACYPI000768	T2	maltase-A1	maltase-like
43	100163994	ACYPI005041	T2	unknown protein	NADPH-dependent diflavin oxidoreductase 1
44	100168922	ACYPI009585	T2	unknown protein	uncharacterized LOC100168922
45	100302479	ACYPI063417	T2	unknown protein	uncharacterized LOC100302479
46	103308568	ACYPI088277	T2	unknown protein	uncharacterized LOC103308568
47	100569669	ACYPI28317	T2	unknown protein	uncharacterized LOC100569669
48	103310452	ACYPI38240	T2	glutathione peroxidase-2	probable phospholipid hydroperoxide glutathione peroxidase
49	100159010	ACYPI000422	T3	unknown protein	apolipophorin
50	100159324	ACYPI000707	T3	unknown protein	centromere-associated protein E
51	100159424	ACYPI000797	T3	unknown protein	zinc finger CCCH domain-containing protein 14-like
52	100160120	ACYPI001445	T3	unknown protein	uncharacterized protein PF11_0213-like
53	100160408	ACYPI001706	T3	similar to Der1-like domain family	derlin-1
54	100162067	ACYPI003247	T3	similar to CG6583-PA	CG6583-like
55	100162155	ACYPI003327	T3	unknown protein	sialin
56	100162451	ACYPI003602	T3	unknown protein	synaptosomal-associated protein 25
57	100162635	ACYPI003780	T3	unknown protein	neurabin-1
58	100163300	ACYPI004394	T3	unknown protein	uncharacterized LOC100163300
59	100163506	ACYPI004591	T3	chromatin STP2	VID27-like protein
60	103311889	ACYPI004866	T3	similar to CG11699-PA	transmembrane protein 242
61	100165162	ACYPI006124	T3	unknown protein	DnaJ-like protein subfamily C member 3
62	100165853	ACYPI006775	T3	similar to CG2471-PA	leucine-rich repeat and death domain-containing protein 1
63	100166123	ACYPI007022	T3	unknown protein	uncharacterized LOC100166123
64	100166523	ACYPI007387	T3	similar to ring finger protein 185	ring finger protein 5-like
65	100169542	ACYPI010151	T3	unknown protein	serine/threonine-protein kinase PRP4 homolog
66	100169561	ACYPI010168	T3	similar to CG5861-PA	transmembrane protein 147
67	103307697	ACYPI071317	T3	zinc-dependent phospholipase C	uncharacterized LOC103307697
68	100163309	ACYPI073648	T3	AHNAK nucleoprotein (desmoyokin)	aminopeptidase N-like
69	100168923	ACYPI080546	T3	glutathione S transferase D10	uncharacterized LOC100168923
70	100573120	ACYPI089376	T3	CG2839	stress response protein NST1-like
71	100166137	ACYPI22506	T3	unknown protein	uncharacterized protein
72	100570990	ACYPI24281	T3	unknown protein	uncharacterized LOC100570990
73	100159447	ACYPI26959	T3	peroxidase	peroxidase-like
74	100302384	ACYPI42782	T3	similar to CG9849-PA	uncharacterized LOC100302384
75	100571995	ACYPI45597	T3	unknown protein	uncharacterized LOC100571995
76	103310026	ACYPI45769	T3	major royal jelly protein (yellow-g2)	uncharacterized LOC103310026
77	100571180	ACYPI46095	T3	unknown protein	uncharacterized LOC100571180
78	100574757	ACYPI48356	T3	unknown protein	uncharacterized LOC100574757
79	103311609	ACYPI48849	T3	unknown protein	zinc finger MYM-type protein 1-like
80	100573202	ACYPI54712	T3	unknown protein	uncharacterized LOC100573202
81	100187582	ACYPI56620	T3	cuticular protein	cuticular protein 28
82	100145855	ACYPI000097	T4	Mp10	chemosensory protein-like
83	100160305	ACYPI001610	T4	Mp30	RR1 cuticle protein 10
84	100160479	ACYPI001774	T4	Mp2	uncharacterized LOC100160479
85	100169619	ACYPI010222	T4	Mp42	uncharacterized LOC100169619
86	100144774	ACYPI000002	T5	sucrase	sucrase
87	100160208	ACYPI001523	T5	chorin peroxidase H6	similar to peroxinectin
88	100161043	ACYPI002298	T5	trehalase (Me5)	trehalase-like
89	100166071	ACYPI006974	T5	cathepsin L	cathepsin L
90	100166170	ACYPI007065	T5	contig_37	stromal cell-derived factor 2-like
91	100166428	ACYPI007300	T5	endoribonuclease	endoribonuclease dcr-1
92	100167383	ACYPI008182	T5	juvenile hormone binding protein homolog	juvenile hormone binding protein-like
93	100168185	ACYPI008911	T5	dipeptidyl carboxypeptidase	angiotensin converting enzyme-like
94	100168963	ACYPI009625	T5	EMP24 like	transmembrane emp24 domain-containing protein 6-like
95	100168332	ACYPI071951	T5	peptidase M1	uncharacterized LOC103307697
96	100165676	ACYPI082770	T5	multicopper oxidase-1 (laccase)	laccase-1-like
97	100164982	ACYPI52702	T5	cathepsin B	cathepsin B-like
98	100165346	ACYPI006300	T6	Me25	carbonic anhydrase 2-like
99	100572792	ACYPI21412	T6	Me20	uncharacterized LOC100572792
100	100574626	ACYPI21663	T6	Me14	lipase member H-B-like
101	100572792	ACYPI53825	T6	Me17	uncharacterized LOC100572792
102	100574728	ACYPI56566	T6	Me13	uncharacterized LOC100574728

**Table 2:** Proteomics-based list of proposed aphid-saliva components.

	Gene ID	Transcript ID	Code	Annotation in Source	Annotation in AphidBase
1	100158673	ACYPI000113	P1	glucose dehydrogenase [FAD quinone]-like	glucose dehydrogenase [FAD quinone]-like
2	100575164	ACYPI000733	P1	dipeptidyl carboxypeptidase	angiotensin-converting enzyme-like
3	100168185	ACYPI008911	P1	dipeptidyl carboxypeptidase	angiotensin converting enzyme-like
4	100168750	ACYPI009427	P1	M1 zinc metalloprotease	aminopeptidase N-like
5	100169243	ACYPI009881	P1	putative sheath protein	uncharacterized LOC100169243
6	100169595	ACYPI010198	P1	unknown protein	aminopeptidase N-like
7	100159063	ACYPI000472	P2	uncharacterized protein	uncharacterized LOC100159063
8	100159632	ACYPI000986	P2	uncharacterized protein LOC100159632 isoform X2	uncharacterized LOC100159632
9	100162450	ACYPI003601	P2	micronuclear linker histone polyprotein-like	micronuclear linker histone polyprotein-like
10	100165393	ACYPI006346	P2	uncharacterized protein	uncharacterized LOC100165393
11	100167863	ACYPI008617	P2	Protein c002	uncharacterized LOC100167863
12	100569515	ACYPI33755	P2	zinc finger protein 853-like	zinc finger protein 853-like
13	100572986	ACYPI56506	P2	uncharacterized protein	uncharacterized LOC100572986
14	100164493	ACYPI56654	P2	3-hydroxyacyl-CoA dehydrogenase type-2	3-hydroxyacyl-CoA dehydrogenase type-2-like
15	100569954	ACYPI009042	P2	similar to alpha-amylase	similar to alpha-amylase
16	100164420	ACYPI005439	P3	PAMP	serine/threonine-protein phosphatase 2A activator-like
17	100166830	ACYPI007670	P3	RNA helicase	putative pre-mRNA-splicing factor ATP-dependent RNA helicase PRP1
18	100168487	ACYPI009182	P3	zinc binding dehydrogenase	reticulon-4-interacting protein 1 homolog, mitochondrial-like
19	100158679	ACYPI000119	P4	disulfide isomerase	endoplasmic reticulum resident protein 44-like
20	100158867	ACYPI000288	P4	glucose dehydrogenase	glucose dehydrogenase [FAD, quinone]-like
21	100160570	ACYPI001857	P4	yellow e-3 like protein	protein yellow-like
22	100161000	ACYPI002258	P4	M1 zinc metalloprotease	endoplasmic reticulum aminopeptidase 2-like
23	100161043	ACYPI002298	P4	trehalase	trehalase-like
24	100161198	ACYPI002439	P4	glutathione peroxidase-1 (Me23)	uncharacterized LOC100161198
25	100161399	ACYPI002622	P4	calreticulin	calreticulin-like
26	100162791	ACYPI003917	P4	SCP GAPR-1	putative uncharacterized protein DDB_G0277255
27	100164598	ACYPI005594	P4	disulfide isomerase	protein disulfide-isomerase A3
28	100166809	ACYPI007650	P4	beta-galactosidase precursor	beta-galactosidase-like
29	100166837	ACYPI007677	P4	calreticulin	calreticulin-like
30	100167188	ACYPI008001	P4	Armet	mesencephalic astrocyte-derived neurotrophic factor homolog
31	100167427	ACYPI008224	P4	uncharacterized protein	uncharacterized LOC100167427
32	100167585	ACYPI008370	P4	CLIP-domain serine protease	uncharacterized LOC100167585
33	100168202	ACYPI008926	P4	disulfide isomerase	protein disulfide-isomerase A6
34	100169107	ACYPI009755	P4	disulfide isomerase	protein disulfide-isomerase
35	100572781	ACYPI23752	P4	carbonic anhydrase II	carbonic anhydrase 7-like
36	100144902	ACYPI000047	P5	ribosomal protein S28e-like	ribosomal protein S28e-like
37	100159010	ACYPI000422	P5	unknown protein	apolipophorin
38	100160057	ACYPI001389	P5	glutamyl aminopeptidase-like	glutamyl aminopeptidase-like
39	100163088	ACYPI004198	P5	unknown protein	similar to apolipophorin
40	100164823	ACYPI005810	P5	similar to AGAP000885-PA, partial	similar to AGAP000885-PA, partial
41	100167066	ACYPI007889	P5	unknown protein	ras-related protein Rab-26
42	100167557	ACYPI008348	P5	unknown protein	ribosomal protein P2-like
43	100167928	ACYPI008675	P5	unknown protein	insulin-degrading enzyme isoform X1
44	100168563	ACYPI009253	P5	unknown protein	60 kDa heat shock protein, mitochondrial
45	100169180	ACYPI009821	P5	fatty acid/phospholipid synthesis protein	fatty acid/phospholipid synthesis protein

**Table 3:** Proposed Pea Aphid Saliva-Proteome

Gene ID	Transcript ID	Code	Annotation	Signal Peptide Probablity	Cleavage Site	Anchor Probability	ER Retention Signal	RPKM
								
100169243	ACYPI009881	T1, P1	putative sheath protein	0.99	25-26	0.00		9654
100167863	ACYPI008617	T1, P2	Protein c002	0.91	23-24	0.05		8610
100569946	ACYPI39568	T1	cysteine rich protein	0.83	28-29	0.04		8217
100165393	ACYPI006346	T1, P2	unknown protein	1.00	19-20	0.00		7564
100167427	ACYPI008224	T1, P4	unknown protein	0.61	27-28	0.14		6113
100159087	ACYPI000490	T1	unknown protein	0.97	22-23	0.00		4361
100159063	ACYPI000472	T1, P2	unknown protein	1.00	26-27	0.00		4326
100570716	ACYPI45001	T1	unknown protein	0.59	28-29	0.29		2989
100166545	ACYPI007406	T1	unknown protein	1.00	22-23	0.00		2703
100160421	ACYPI001719	T1	unknown protein	0.98	18-19	0.02		2494
100569515	ACYPI33755	P2	zinc finger protein 853-like	1.00	19-20	0.00		2154
100302479	ACYPI063417	T2	unknown protein	0.90	24-25	0.05		2055
100159644	ACYPI49603	T1	protein sorting-associated protein 53	0.60	24-25	0.00		1875
100159932	ACYPI001271	T1	unknown protein	1.00	23-24	0.00	KEDK	1754
100160301	ACYPI001606	T1	unknown protein	0.98	24-25	0.00		1279
100160906	ACYPI002172	T1	trichohyalin like	0.54	25-26	0.25		1237
100160226	ACYPI001541	T1	unknown protein	0.80	27-28	0.16		1027
100167919	ACYPI008667	T1	unknown protein	0.94	28-29	0.02		892
100158867	ACYPI000288	T1, P4	glucose dehydrogenase [FAD,quinone]-like	0.94	24-25	0.04		881
100164831	ACYPI005818	T1	unknown protein	1.00	21-22	0.00		695
100164982	ACYPI52702	T5	cathepsin B-like	1.00	20-21	0.00		666
100572986	ACYPI56506	P2	unknown protein	0.81	28-29	0.04		611
100159750	ACYPI001099	T1	unknown protein	0.98	22-23	0.01		587
100161198	ACYPI002439	T1, P4	glutathione peroxidase-1 (Me23 ortholog)	0.91	28-29	0.02		580
100159632	ACYPI000986	T1, P2	unknown protein	0.98	22-23	0.00		562
100159010	ACYPI000422	T3, P5	apolipophorins	1.00	19-20	0.00		545
103310452	ACYPI38240	T2	phospholipid hydroperoxide glutathione peroxidase	0.53	18-19	0.18		357
100145855	ACYPI000097	T4	chemosensory protein-like	1.00	22-23	0.00		354
100160479	ACYPI001774	T4	Mp2 ortholog	1.00	20-21	0.00		290
100167383	ACYPI008182	T5	juvenile hormone binding protein-like	0.99	20-21	0.00		269
100161399	ACYPI002622	P4	calreticulin	1.00	23-24	0.00	HDEL	253
100144902	ACYPI000047	P5	ribosomal protein S28e-like	0.57	21-22	0.00		132
100168118	ACYPI55147	T1	nucleolar protein of 40 kDa	1.00	18-19	0.00		130
100163300	ACYPI004394	T3	unknown protein	0.91	29-30	0.09		110
100163506	ACYPI004591	T3	VID27-like protein	0.99	28-29	0.00		98
100187582	ACYPI56620	T3	cuticular protein 28 (cp28)	1.00	18-19	0.00		96
100160601	ACYPI001887	T1	unknown protein	0.76	20-21	0.20		86
100144774	ACYPI000002	T5	sucrase (S1)	0.95	21-22	0.00		84
100162791	ACYPI003917	T1, P4	unknown protein	1.00	23-23	0.00		76
100162547	ACYPI003695	T1	unknown protein	0.83	19-20	0.00		75
100574994	ACYPI56502	T1	unknown protein	0.73	28-29	0.15		73
100169287	ACYPI009919	T1	unknown protein	0.85	22-23	0.05		73
100162584	ACYPI55148	T1	chondroitin sulfate proteoglycan 4	1.00	18-19	0.00		66
100166071	ACYPI006974	T5	cathepsin L (Ctsl)	1.00	19-20	0.00		62
100169107	ACYPI009755	P4	protein disulfide-isomerase	1.00	18-19	0.00	KDEL	58
100161043	ACYPI002298	T5, P4	trehalase	0.97	20-21	0.00		58
100302326	ACYPI43360	T1	unknown protein	1.00	22-23	0.00		55
100168563	ACYPI009253	P5	60 kDa heat shock protein, mitochondrial	0.59	18-19	0.00		54
100159485	ACYPI000852	T1	unknown protein	1.00	25-26	0.00		51
100162450	ACYPI003601	T1, P2	micronuclear linker histone polyprotein-like	0.99	19-20	0.01		50
100575164	ACYPI000733	T1, P1	dipeptidyl carboxypeptidase	0.99	18-19	0.00		47
100161000	ACYPI002258	P4	endoplasmic reticulum aminopeptidase 2	1.00	19-20	0.00		45
100159160	ACYPI000558	T1	unknown protein	0.98	25-26	0.01		43
100164598	ACYPI005594	P4	protein disulfide isomerase A3	1.00	20-21	0.00	KHEL	40
100160305	ACYPI001610	T4	cuticle protein 10 (cprr1-10)	1.00	18-19	0.00		40
100168332	ACYPI071951	T5	similar to alpha-amylase	0.83	28-29	0.16		36
100161690	ACYPI002891	T1	cadherin	0.96	18-19	0.02		36
100169619	ACYPI010222	T4	Mp42 ortholog	0.51	22-23	0.45		34
100166123	ACYPI007022	T3	unknown protein	1.00	21-22	0.00		31
100165853	ACYPI006775	T3	Leu-rich repeat-containing protein 57	1.00	18-19	0.00		31
100165676	ACYPI082770	T5	laccase-1	0.99	27-28	0.70		31
100169595	ACYPI010198	P1	aminopeptidase N-like	0.98	23-24	0.02		30
100158679	ACYPI000119	P4	endoplasmic reticulum resident protein 44	0.75	32-33	0.08	KEEL	29
100574626	ACYPI21663	T6	lipase member H-B like (Me14 ortholog)	0.75	19-20	0.13		29
100160408	ACYPI001706	T3	derlin like protein	0.72	32-33	0.04		29
100167188	ACYPI008001	T1, P4	Armet (MANF)	0.99	20-21	0.01		28
100162451	ACYPI003602	T3	synaptosomal-associated protein 25	0.92	18-19	0.00		27
100571995	ACYPI45597	T3	unknown protein	1.00	20-21	0.00		26
100160554	ACYPI001843	T1	serine/threonine-protein kinase like	0.92	25-26	0.01		25
100167557	ACYPI008348	P5	ribosomal protein P2-like	0.94	19-20	0.03		24
100168922	ACYPI009585	T2	unknown protein	0.96	20-21	0.00		24
100158673	ACYPI000113	P1	glucose dehydrogenase (FAD,quinone]-like	0.97	21-22	0.00		23
100159805	ACYPI001152	T1	unknown protein	1.00	23-24	0.00		22
100574728	ACYPI56566	T6	Me13 ortholog	1.00	22-23	0.00		21
100572792	ACYPI21412	T6	Me20 ortholog	1.00	25-26	0.00		20
100168202	ACYPI008926	P4	protein disulfide-isomerase A6	1.00	18-19	0.00	KEEL	20
100569954	ACYPI009042	P2	maltase 2	0.99	21-22	0.00		19
100167585	ACYPI008370	P4	CLIP-domain serine protease	0.94	19-20	0.03		19
100159394	ACYPI000768	T2	maltase like	0.90	20-21	0.00		16
100166523	ACYPI007387	T3	ring finger protein 5-like	0.80	18-19	0.00		15
100166702	ACYPI007553	T1	unknown protein	0.96	22-23	0.01		15
100570990	ACYPI24281	T3	unknown protein	0.96	25-26	0.02		14
100164823	ACYPI005810	P5	AGAP000885-PA-like	0.99	27-28	0.01		13
103311889	ACYPI004866	T3	transmembrane protein 242-like	0.92	50-51	0.48		13
100167928	ACYPI008675	P5	insulin-degrading enzyme like	0.99	24-25	0.00		12
100165162	ACYPI006124	T3	dnaJ homolog subfamily C member-3	0.95	28-29	0.01		12
100169561	ACYPI010168	T3	transmembrane protein 147-like (Tmem147)	0.98	23-24	0.02		12
100164420	ACYPI005439	P3	Ser/Thr-protein phosphatase 2A activator-like	1.00	24-25	0.00		12
100162155	ACYPI003327	T3	sialin	0.89	43-44	0.24		11
100162067	ACYPI003247	T3	CG6583 like	1.00	23-24	0.00		11
100169180	ACYPI009821	P5	solute carrier family 25 member 36	0.81	24-25	0.11		11
100302384	ACYPI42782	T3	unknown protein	0.55	29-30	0.43		11
100168487	ACYPI009182	P3	reticulon-4-interacting protein 1 homolog	1.00	20-21	0.00		11
100168923	ACYPI080546	T3	unknown protein	0.78	28-29	0.77		10
100166830	ACYPI007670	P3	pre-mRNA-splicing factor RNA helicase PRP1-like	1.00	19-20	0.00		10
100166170	ACYPI007065	T5	stromal cell-derived factor 2-like	0.92	25-26	0.06	HTEL	10
100160208	ACYPI001523	T5	peroxinectin like	1.00	34-35	0.00		10
100161239	ACYPI002476	T1	3'(2'),5'-bisphosphate nucleotidase 1-like	0.94	19-20	0.00		10
100573202	ACYPI54712	T3	unknown protein	0.98	19-20	0.00		8.9
100159324	ACYPI000707	T3	myosin-J heavy chain	0.98	31-32	0.00		8.7
100165346	ACYPI006300	T6	carbonic anhydrase 2 (Me25 ortholog)	1.00	23-24	0.00		7.7
100163309	ACYPI073648	T3	aminopeptidase N-like	0.80	17-18	0.00		7.3
100169542	ACYPI010151	T3	Ser/Thr-protein kinase PRP4 homolog	0.88	25-26	0.00		7.0
100168963	ACYPI009625	T5	emp24 domain-containing protein 6-like	0.94	39-40	0.06		6.9
100168750	ACYPI009427	P1	aminopeptidase N	0.99	19-20	0.00		6.4
103307697	ACYPI071317	T3	zinc-dependent phospholipase C	0.90	15-16	0.00		6.2
100164493	ACYPI56654	P2	3-hydroxyacyl-CoA dehydrogenase type-2	1.00	32-33	0.01		5.6
100573120	ACYPI089376	T3	stress response protein NST1 like	0.91	25-26	0.07		5.6
100574757	ACYPI48356	T3	unknown protein	0.60	18-19	0.00		5.2
100167066	ACYPI007889	P5	ras-related protein Rab-27	0.60	22-23	0.00		5.1
100166428	ACYPI007300	T5	endoribonuclease dcr-1	1.00	21-22	0.00		5.1
100163994	ACYPI005041	T2	NADPH-dependent diflavin oxidoreductase 1	0.90	18-19	0.01		5.1
100571180	ACYPI46095	T3	unknown protein	0.50	24-25	0.26		4.2
100159447	ACYPI26959	T3	peroxidase	1.00	19-20	0.00		4.0
100168185	ACYPI008911	T5, P1	dipeptidyl carboxypeptidase	0.99	25-26	0.01		3.3
103310026	ACYPI45769	T3	major royal jelly protein (yellow-g2) protein 1-like	0.52	16-17	0.00		3.0
100159424	ACYPI000797	T3	Zn-finger CCCH domain-containing protein 14	0.89	18-19	0.00		2.7
100569669	ACYPI28317	T2	unknown protein	0.96	26-27	0.02		2.0
100160120	ACYPI001445	T3	protein PF11_0213 like	0.89	18-19	0.00		2.0
100166809	ACYPI007650	P4	beta-galactosidase like	1.00	23-24	0.00		1.5
100162635	ACYPI003780	T3	neurabin-1	0.82	31-32	0.00		1.3
100160057	ACYPI001389	P5	unknown protein	0.88	26-27	0.07		1.1
100572792	ACYPI53825	T6	Me17 ortholog	1.00	24-25	0.00		0.7
100574100	ACYPI38795	T1	thrombin-like enzyme cerastocytin	0.99	16-17	0.00		0.5
100572781	ACYPI23752	P4	carbonic anhydrase 7	1.00	21-22	0.00		0.4
100160570	ACYPI001857	P4	protein yellow-like	0.87	32-33	0.13		0.3
103311609	ACYPI48849	T3	zinc finger MYM-type protein 1 like	0.65	18-19	0.00		0.3
100166137	ACYPI22506	T3	N-alpha-acetyltransferase 35 NatC auxiliary subunit	0.80	36-37	0.00		0.3
100166837	ACYPI007677	P4	calreticulin-like	1.00	23-24	0.01		0.1
100163088	ACYPI004198	T1, P5	apolipophorins	1.00	19-20	0.00		0.1
103308568	ACYPI088277	T2	unknown protein	0.91	18-19	0.00		0

**Table 4:** tBLASTx matches between pea aphid saliva-proteome components and *Bemisia* transcripts

Pea Aphid Gene ID	Pea aphid NCBI annotation	NCBI ID aphid transcript	NCBI ID whitefly top-hit transcript	log (e-value)	Bemisia NCBI annotation
100145855	chemosensory protein-like	NM_001126180.2	KT694345.1	−35	chemosensory protein 2
100144774	sucrase (S1)	NM_001126135.2	XM_019053092.1	(0)	maltase 2-like
103308568	unknown protein	XM_008182173.1	XM_019052646.1	−168	uncharacterized LOC109037822
100164982	cathepsin B-like	NM_001246084.1	XM_019045930.1	−70	cathepsin B-like
100159644	protein sorting-associated protein 53	XM_001944835.4	XM_019055223.1	(0)	vacuolar protein sorting-associated protein 53
100158867	glucose dehydrogenase [FAD, quinone]-like	XM_001943360.4	XM_019045136.1	−109	glucose dehydrogenase [FAD, quinone]-like
100167383	juvenile hormone binding protein-like	NM_001204960.1	XM_019054771.1	−102	protein takeout-like
100569954	maltase 2	XM_003246839.3	XM_019040276.1	−177	maltase A1-like
100144902	ribosomal protein S28e-like	NM_001126197.2	XM_019049779.1	−24	40S ribosomal protein S28
100159010	apolipophorins	XM_008185731.2	XM_019048511.1	−159	apolipophorins
100161399	calreticulin	XM_003240040.3	XM_019042469.1	−178	calreticulin
100159632	unknown protein	XM_001949497.3	XM_019055567.1	−91	glucose dehydrogenase [FAD, quinone]-like
103310452	phospholipid hydroperoxide glutathione peroxidase	NM_001293432.1	XM_019061104.1	−70	glutathione peroxidase-2
100161198	glutathione peroxidase-1 (Me23 ortholog)	NM_001162003.2	XM_019050639.1	−18	lipid hydroperoxide glutathione peroxidase
100168118	nucleolar protein of 40 kDa	XM_001946803.4	XM_019045258.1	−52	nucleolar protein of 40 kDa-like
100187582	cuticular protein 28 (cp28)	NM_001134289.1	XM_019054865.1	−15	larval cuticle protein A2B-like
100162791	unknown protein	XM_001948421.4	XM_019047663.1	−23	uncharacterized LOC109034480
100162584	chondroitin sulfate proteoglycan 4	XM_001951082.4	XM_019051208.1	(0)	chondroitin sulfate proteoglycan 4
100166071	cathepsin L (Ctsl)	NM_001135938.1	XM_019055808.1	−140	cathepsin L1
100169107	protein disulfide-isomerase	XM_008184943.2	XM_019056804.1	−177	protein disulfide isomerase
100161043	trehalase	XM_001950229.4	XM_019047997.1	(0)	trehalase-like
100168563	60 kDa heat shock protein, mitochondrial	XM_016801063.1	XM_019053486.1	(0)	60 kDa heat shock protein, mitochondrial
100575164	dipeptidyl carboxypeptidase	XM_003242835.3	XM_019052809.1	(0)	angiotensin-converting enzyme-like
100161000	endoplasmic reticulum aminopeptidase 2	XM_008181654.2	XM_019050840.1	(0)	aminopeptidase N
100164598	protein disulfide isomerase A3	XM_001950371.4	XM_019051140.1	(0)	protein disulfide-isomerase A3
100160305	cuticle protein 10 (cprr1-10)	NM_001161959.2	XM_019041783.1	−31	cuticle protein 3-like
100168332	similar to alpha-amylase	ACYPI009042	XM_019040276.1	(0)	maltase A1-like
100166123	unknown protein	NM_001162762.2	XM_019048611.1	−54	uncharacterized LOC109035114
100165853	Leu-rich repeat-containing protein 57	XM_001943902.4	XM_019041438.1	−48	leucine-rich repeat protein soc-2
100165676	laccase-1	XM_003241838.2	XM_019057635.1	−114	laccase-2
100169595	aminopeptidase N-like	XM_001948813.4	XM_019052513.1	(0)	aminopeptidase N
100158679	endoplasmic reticulum resident protein 44	XM_001951459.4	XM_019042102.1	−160	endoplasmic reticulum resident protein 44
100574626	lipase member H-B like (Me14 ortholog)	XM_003246187.3	XM_019056906.1	−57	lipase member H-B-like
100160408	derlin like protein	NM_001293469.1	XM_019060915.1	−111	derlin-1
100167188	Armet (MANF)	XM_001949506.4	XM_019059664.1	−36	MANF homolog
100162451	synaptosomal-associated protein 25	XM_008190415.2	XM_019046121.1	−127	synaptosomal-associated protein 25
100167557	ribosomal protein P2-like	NM_001171963.1	XM_019055463.1	−23	60S acidic ribosomal protein P2
100158673	glucose dehydrogenase [FAD, quinone]-like	XM_001946910.4	XM_019055567.1	−98	glucose dehydrogenase [FAD, quinone]-like
100168202	protein disulfide-isomerase A6	XM_001948232.4	XM_019059175.1	(0)	protein disulfide isomerase A6
100159394	maltase like	XM_001943282.4	XM_019061713.1	(0)	maltase 2-like
100166523	ring finger protein 5-like	NM_001162239.1	XM_019049104.1	−60	E3 ubiquitin-protein ligase RNF185-like
100164823	AGAP000885-PA-like	XM_001947290.1	XM_019052513.1	−175	aminopeptidase N
103311889	transmembrane protein 242-like	NM_001293338.1	XM_019061249.1	−38	transmembrane protein 242
100167928	insulin-degrading enzyme like	XM_008182127.2	XM_019052714.1	(0)	insulin-degrading enzyme
100165162	dnaJ homolog subfamily C member 3	XM_001948989.4	XM_019061914.1	−179	dnaJ homolog subfamily C member 3
100169561	transmembrane protein 147-like (Tmem147)	NM_001162359.1	XM_019045265.1	−75	transmembrane 147
100164420	Ser/Thr-protein phosphatase 2A activator-like	NM_001162153.1	XM_019056931.1	−109	Ser/Thr-protein phosphatase 2A activator-like
100162155	sialin	XM_001947103.4	XM_019053745.1	(0)	sialin-like
100162067	CG6583 like	NM_001162039.2	XM_019058779.1	−55	uncharacterized LOC109042173
100169180	solute carrier family 25 member 36	XM_001951878.4	XM_019057637.1	−130	solute carrier family 25 member 36
100302384	unknown protein	NM_001162978.1	XM_019043661.1	−43	PRADC1-like protein
100168487	reticulon-4-interacting protein 1 homolog	XM_001948134.4	XM_019042511.1	−88	reticulon-4-interacting protein 1 homolog
100168923	unknown protein	XM_016806800.1	XM_019056630.1	−53	uncharacterized LOC109040643
100166830	pre-mRNA-splicing factor RNA helicase PRP1-like	XM_001945136.4	XM_019052488.1	(0)	pre-mRNA-splicing factor RNA helicase PRP1
100166170	stromal cell-derived factor 2-like	NM_001162218.2	XM_019051409.1	−65	stromal cell-derived factor 2
100160208	peroxinectin like	XM_001947501.1	XM_019045246.1	−46	peroxidase-like
100161239	3′(2′),5′-bisphosphate nucleotidase 1-like	NM_001162636.2	XM_019054567.1	−94	3′(2′),5′-bisphosphate nucleotidase 1
100159324	myosin-J heavy chain	XM_001950688.4	XM_019047277.1	−122	myosin 11-like
100165346	carbonic anhydrase 2 (Me25 ortholog)	XM_001945697.4	XM_019051605.1	−101	carbonic anhydrase 2-like
100169542	Ser/Thr-protein kinase PRP4 homolog	XM_008185390.2	XM_019043808.1	−132	Ser/Thr-protein kinase PRP4 homolog
100168963	emp24 domain-containing protein 6-like	NM_001246005.2	XM_019040879.1	−101	emp24 domain-containing protein B
100168750	aminopeptidase N	XM_001944729.4	XM_019052513.1	(0)	aminopeptidase N
100164493	3-hydroxyacyl-CoA dehydrogenase type-2	NM_001246102.2	XM_019056361.1	−89	3-hydroxyacyl-CoA dehydrogenase type-2
100167066	ras-related protein Rab-27	XM_016803815.1	XM_019053904.1	−98	ras-related protein Rab-37-like
100166428	endoribonuclease dcr-1	XM_008189326.2	XM_019044285.1	(0)	endoribonuclease Dicer
100163994	NADPH-dependent diflavin oxidoreductase 1	XM_008180762.2	XM_019051309.1	(0)	NADPH-dependent diflavin oxidoreductase 1
100159447	peroxidase	XM_008188105.1	XM_019045267.1	−79	peroxidase-like
100168185	dipeptidyl carboxypeptidase	NM_001135912.1	XM_019052809.1	(0)	angiotensin-converting enzyme-like
103310026	major royal jelly protein (yellow-g2) protein 1-like	XM_008187022.2	XM_019056708.1	−26	major royal jelly protein 1-like
100166809	beta-galactosidase like	XM_001948514.3	XM_019060401.1	−163	beta-galactosidase-like
100162635	neurabin-1	XM_003241864.3	XM_019042683.1	(0)	uncharacterized LOC109031256
100160057	unknown protein	XM_016806012.1	XM_019042907.1	(0)	glutamyl aminopeptidase-like
100572781	carbonic anhydrase 7	XM_008190777.2	XM_019043651.1	−56	carbonic anhydrase 3-like
100160570	protein yellow-like	XM_016801442.1	XM_019060874.1	−77	protein yellow-like
103311609	zinc finger MYM-type protein 1 like	XM_008191282.1	XM_019052267.1	−29	uncharacterized LOC109037544
100166137	N-alpha-acetyltransferase 35 NatC auxiliary subunit	XM_001952176.4	XM_019047618.1	(0)	acetyltransferase 35 NatC auxiliary subunit
100166837	calreticulin-like	XM_001944020.4	XM_019042469.1	−170	calreticulin
100163088	apolipophorins	XM_001949101.1	XM_019048511.1	−60	apoipophorins
