# Supplementary material for: Toward a Comprehensive Pea Aphid Saliva-Proteomewith Insights from Transcripts from the Whitefly Bemisia tabaci
Source: Biochem Mol Biol J. Author manuscript; Available in PMC 2026 Jun 12. (PMC13256243)
Supplement: alignments [file NIHMS2167181-supplement-alignments.docx]

**SUPPLEMENTARY TABLE I:** Alignment of several pairs of pea aphid and *Bemisia tabaci* proteins in Table 4. Ap indicates pea aphid; Bt indicates *Bemisia tabaci*.

**Unknown protein (pea aphid) and glucose dehydrogenase (*Bemisia*).** The first 500 residues of the predicted pea aphid protein (encoded by gene 100159632) are not aligned and are therefore not shown here. e-Value = 10^(-91).

ApUnknown 501 DDDDKPDDNDSENDDSEEEETSEDEVSVYEYLNTF---RKFRFDSGLNSV 547

....::|...|....::|....|.: |.:.:....|.:

BtGlucDehy 1 MRVQNTELYPTFFSIIAVVLVTNNYWFVRAYHYSLDRNKL 40

ApUnknown 548 GII--GEVL-SQLDYQRYKL---MDDSI-----YPQDSTRYVINDEEFDF 586

..| .||. |.:|..:.:| .::.| |.:|..|.:.|.|||||

BtGlucDehy 41 RTIQADEVTGSMVDGFKMELQIHQEEWIETARHYFEDYNRNLTNGEEFDF 90

ApUnknown 587 IIVGGGNAGCVLANKLSENVKWKVLLIEAGGDPFPITQIPSLWDRSLNSV 636

||||.|:||.|:||:||.|..|:|||||||.||..::::|:::.....:.

BtGlucDehy 91 IIVGAGSAGSVVANRLSANENWRVLLIEAGDDPPLLSEVPAMYSALQGTT 140

ApUnknown 637 ADWQFKIQPDSTTGFGIGGNM-KIHKGRGLGGSSITSAQLYVRGSEQLYN 685

||||:....|:.|..|:.... .:.||:.|||:|..:...||||:.:.|:

BtGlucDehy 141 ADWQYVTAKDNQTCLGLKNQQCSLPKGKLLGGTSSINGMFYVRGNRKDYD 190

ApUnknown 686 SLVKKGLKNWSYNTTETYFKKVERIRSI-TKTETNTTIY-GKCGLIPVSK 733

:...:|...|::..|..||||.|.:.:: .|.:.....| |..|.:.|..

BtGlucDehy 191 AWESQGNSGWNHRETLKYFKKSENVENVYIKADDGLMKYHGTGGYLSVDS 240

ApUnknown 734 FRKTEVSVLEKIVCSGFEHIGCKKESDINEKDIEVGFVSMQGIIKNGRSI 783

|..|...:.:..:.:..| :|....:.:| .:.:.|:..:...:..||..

Bemisia 241 FNSTPHYLADHYISAAAE-LGYGLLTCVN-GETQTGYSFLSATVSQGRRC 288

ApUnknown 784 NTAKAYLSPIFGRENLKVMKYSRVTKIIVNKTEMKATGVEVQTKFGQTLT 833

:.|||:|.|...|:||||.....||||::.:...||.|||:.. :.:|:.

BtGlucDehy 289 SAAKAFLKPARDRDNLKVSTERTVTKILLRQKSKKAYGVELSV-YDRTVR 337

ApUnknown 834 IKAKLEVLLCAGAVGSAQILLASGIGPKKHLSEMEVPVVKDLKVGENFLI 883

:.||.||:|.||::||.|:::.||||||.||.||.|..::||.||:| |.

BtGlucDehy 338 VHAKKEVILSAGSIGSPQLMMVSGIGPKSHLEEMGVDFIQDLPVGKN-LQ 386

ApUnknown 884 TPVFTGFVISYDKSVVCNQTD----EEIAFKYLARHSGPLSRPNGMSFGG 929

..|....|::|:..:: |:|| .:..:::|.:.||||:.....||.|

BtGlucDehy 387 DHVALPIVVAYNDQLI-NKTDCLDAYDSFYQFLRKSSGPLTNLGFNSFTG 435

ApUnknown 930 FLNTGMSGSSFADIEVHQFYIPKNSYSKLCQLKSMFGFSDNLLSVYAKLN 979

|::| .....:.||::|.|...||....|......:||.::......::|

BtGlucDehy 436 FIST-TETKDYPDIQIHHFTFAKNDSKALGTFLKGYGFLEDTEKSVLEIN 484

ApUnknown 980 YERAISIFTIALINVKSTSKILLRSKNPLDSPIIIGNMLTEKHDIKSFLE 1029

.:..:::...||:..||..:|||:|.|..|.||||...|.:..|:...::

BtGlucDehy 485 SKSDVTLVIPALLRPKSRGQILLQSANVTDKPIIISGFLNDTDDVAKLID 534

ApUnknown 1030 AIKLLSKIEKSDGMNLVNAKLEDIDLDGCAKYTKKTNEHWECLLKYMVST 1079

|||.:.|..|::.:..:.|:::.|::.||.....:::::|:|:|.:|.|:

BtGlucDehy 535 AIKFVQKFVKTESLTRIGAEIKRINMKGCEAEEFQSDKYWKCMLHHMASS 584

ApUnknown 1080 TSSTAGSCRMGLETDTDAVVDGELNVIGISNLRAVGRSVLPMITSAYSHV 1129

...|.|:|:||.::|..||||..|.|.||..||.|..|::|.|.|..::.

BtGlucDehy 585 LYHTVGTCKMGPKSDPTAVVDHSLKVHGIDRLRVVDASIMPTIVSGNTNA 634

ApUnknown 1130 PCIMVAERAYGMIKSKYN------------ 1147

..:|:.|:|..||..::.

BtGlucDehy 635 AVMMIGEKAADMILHRWRDNNSEKRRKNKN 664

**Cuticle Protein 28-like protein from pea aphid and *Bemisia tabaci.*** e-Value = 10^(-15).

ApCP28 1 MSAKLIIFAACAVATALAVYKEPSYPSYPAAPAY---PAPAYPAAPAYSA 47

:|....:|||.:|| |...| |.|. |.|.||:|

BtCP28 1 MITQLISVATLMAV----------ARAGYLGDPGPG-PLAHAYAA 34

ApCP28 48 PAYPSAPAYPSAPAYPAAPAYPAAPAYPAAPAYSAAPAYKPASYAAPKPY 97

| :.:..|||......||||....|..|:.|.|..||| |.:|:||..:

BtCP28 35 P-FLAGHAYPGFRYAAAAPAPAPPPPKPSFPVYKPAPA--PIAYSAPLVH 81

ApCP28 98 APE---PAYSAPSP-YNFDYSVHDTYTGDIKSQNEYADANGYVKGSYSLV 143

||: |||....| |||||||||.||||.|||:|..|.: .|:||||||

BtCP28 82 APKAYAPAYPDAYPKYNFDYSVHDGYTGDTKSQHETRDGD-VVQGSYSLV 130

ApCP28 144 EPDGSKRTVEYTADDYNGFNAEVKK---EGGYPAPAYSAPAPAYKPAPAP 190

||||:.|||.|.||..|||||.|:: .|...||...||| |.|.|

BtCP28 131 EPDGTLRTVNYAADPVNGFNAVVERSPPHGHAHAPKLYAPA----PGPGP 176

ApCP28 191 YKPAY 195

:.|.:

BtCP28 177 HLPYHG 182

**Unknown protein from pea aphid and *Bemisia tabaci.***  The pea aphid sequence is encoded by gene 100162791. e-Value = 10^(-23).

ApUnk 1 MAKPIYFSLLLLVLCLIYESYGNHHTVRHIRLPSHRISSDNGPYVSS-SS 49

.|...:| ||

BtUnk 1 MGTKTASWSS 10

ApUnk 50 SSSSSTTGADATATSESAETSLSPSSSLRKSLSLNNSLGYGKNRGLGLNS 99

.|...:.|:.:.::|.|:|.||| ..|....||..::|.|

BtUnk 11 VSGGMSVGSRSNSSSSSSEFSLS-GRSASSGLSYPSALSY---------- 49

ApUnk 100 KSLSGKYYEQQPNDMDSDDLNNRQIQNSMNSHFLNNKPQNGVFYKNNGID 149

.|..::..:..:|.::| |

BtUnk 50 ---------------PSASMDLTKFHDSAHAH-----------------D 67

ApUnk 150 RQNSMTSEVLNSKPLKDLYSMNNQNDKTLEGPSIGKRQNSITSGALNFPN 199

.|..::..|:..|..:..|...: |..:||.| .::|...:....

BtUnk 68 FQQPLSRIVMVRKMEQRTYVAAS---KQGQGPKI----ETLTKETVQTFR 110

ApUnk 200 SSKTKIIPLASLMARQKTLSPSSNEDDSQQISVGNDNSSMMMNSIKIPLN 249

.:|| ::.::.|...|.|.:| |:....::..|

BtUnk 111 GNKT-----------ERKVTTSEKRDVSAEI--GDAAKELISLS------ 141

ApUnk 250 EQHIVFKQLRKCCINRECRILQDGEECSINDYFKNMKTKQQPNNPTFTTS 299

|..|.:.|..: :.|..::|.:..:::.

BtUnk 142 ---------RSTCSSPELTV--------------DPKGGRRPLSRCWSSG 168

ApUnk 300 SNGENGLNDMMSKFKLFGKNSFNNLGDDMDDLQETGKPTNMRLGSMRYSA 349

|...:| .|| .:|.:.|..|......:| ||

BtUnk 169 SGSGSG----SSK-----SSSLSRLATDTSSTHSSG--TN---------- 197

ApUnk 350 IIPSIGSSNGMGSYVMPQKRKPINYPHSDPIDLSEDEANEIRDQVLQKSN 399

:|.::|.:.: ...|| ::|.....||..|

BtUnk 198 ---TINTTNTINT------TNTIN-------------SSEFAKLCLQAHN 225

ApUnk 400 FYREKYGLEPFTLDDQLNNCAQDWANQMVKLKIFDHREDNVYGENLFSSL 449

.||.|:.:.|..|:.:|...:::||..:....|.:||:::.||:|:|.|.

BtUnk 226 EYRAKHHVPPLKLNKKLCKYSEEWAKHLASKGILEHRQNSAYGQNIFCSW 275

ApUnk 450 D----FNNLGEQAVDSWYNEITKFNIADEEPELGDNIATHHMTQLLWKSS 495

. ....|::.|||||.|| ||:...:|| .::.:.|.:|::|.:|

BtUnk 276 SSAPRHTVCGQEPVDSWYEEI-KFHPFGKEP---SSLKSGHFSQVVWLTS 321

ApUnk 496 TKLGVGVSKSSNGMYNVVANYDPSGNVRGFFKDNLPE------------- 532

.:|||||::|.||...|||||||.||..|.|.:|:|.

BtUnk 322 AELGVGVARSRNGQIFVVANYDPPGNFLGQFSENVPPLGGFPKFSEDYEK 371

ApUnk 533 --IKQED----IEEAMDSHNSQTISE--PKSI------------------ 556

:||.| .:||:|.||...... ||.|

BtUnk 372 SLLKQYDAKTFTKEALDIHNEYRCKHHVPKLILSKKLCDYAKEWALILVK 421

ApUnk 557 -----------------SWSSSSIPPLES--EWQYE-PYYN--------- 577

.|||..:...:: :|..| ..||

BtUnk 422 DEKLSHRDSRYGENIFSMWSSGRVTAKDACEKWYEEGKDYNYSVEPRNLK 471

BtUnk 472 SGNFTQMIWKSSKEVGFAMIRGKSGRVVIVANYHPAGNISGQFIENVLKP 521

**Trehalase from pea aphid and *Bemisia tobaci.*** e-Value = “0”.

ApTrehalase 1 MRITNLLVVCLAHFAYYTHANNQEFVHLARGYYHVS---NGLQA------ 41

....:|....::|....:.:| |.|.|

BtTrehalase 1 MIFESNRTAKMNLFSSIFFLSNVYNSLNAMQLKDM 35

ApTrehalase 42 -------SCQSQIYCESDLLKDVQLAHIFPDSKTFVDMKLKYSESEILKN 84

||.|::||:|:||.|||:|.::||||.|||.|:||:||..|:.

BtTrehalase 36 YYTSYLPSCDSKVYCDSELLHDVQMARLYPDSKEFVDKKMKYNESYTLEK 85

ApTrehalase 85 YQVLKDGNNGVVP-KEKIVKFVDEHFMDGDELEVWTPSDFNESPSIANRI 133

|:.||..|....| ||::.|||.|||.||:|||||.|.||.|..|:..||

BtTrehalase 86 YEELKIQNGRKTPSKEQLQKFVYEHFEDGNELEVWVPPDFKEHISLEERI 135

ApTrehalase 134 KDKNYKQWALGLNQVWKTLARKVKDDVRLHPDRYSLIWVPNGFAIPGGRF 183

:|..||::|.||||:||.||||||.:|:...|:|||::.||||.||||||

BtTrehalase 136 RDTEYKKFANGLNQIWKILARKVKVEVKEKSDQYSLLYTPNGFCIPGGRF 185

ApTrehalase 184 RELYYWDTYWIVNGMLLCDMSTTARGVIDNILSLVLQFGFMPNGGRVYYL 233

||||||||||||||::|.||..||||||:||:.||.::|.||||.||||.

BtTrehalase 186 RELYYWDTYWIVNGLILSDMVETARGVIENIVYLVNKYGIMPNGARVYYQ 235

ApTrehalase 234 NRSQPPMVTLMVSSYYKATNDFEYVKKVISILDSEFEFWTENRMVTFEKN 283

.|||||.:.||..||||||:|.::|:|.:..|.||||:|..||.|.|.|:

BtTrehalase 236 RRSQPPFLILMFESYYKATSDSKFVEKHLKTLTSEFEWWQNNRKVKFVKD 285

ApTrehalase 284 GKSYTMARYYAPSRGPRPESYREDYESAEFLKTENEKQELYTQIKSAAET 333

.|.|.|.||:|||.||||||||||||.||.:.||.|::..|.::||.||:

BtTrehalase 286 NKRYEMYRYFAPSNGPRPESYREDYELAESIDTEIERERWYGRMKSGAES 335

ApTrehalase 334 GWDFSSRWFITANGSDRGILADIKTTYIIPVDLNCILHKNALLLSSWYSK 383

||||||||||..||...|.|.|:.|..::..|||.::||||:.||.|:.|

BtTrehalase 336 GWDFSSRWFINKNGGYNGTLLDVNTPSLVVTDLNAVMHKNAVFLSEWWGK 385

ApTrehalase 384 MGDTTKAEKYRAIAEKLVYSIQEVMWRPDLGAWFDWDMLNNKSREYFFVS 433

|||..:::.|:.:|:|.:.||:||:|....|.|||:|:.:.|.|:.|:.|

BtTrehalase 386 MGDKYRSKLYKEVADKQLASIEEVLWNEQRGCWFDYDLKSQKPRDQFYPS 435

ApTrehalase 434 NIVPLWTESYNMPKKAVASSVLGYLRDHHIIEADYTVNFNGTPTSLYNSS 483

|..||||.||..||..||:.::.|::...:|..:.|..::|.|.|.:.|.

BtTrehalase 436 NFAPLWTGSYTKPKIQVATRIIEYVKQEEMITNELTARYHGQPCSKFESG 485

ApTrehalase 484 QQWDFPNAWPPLQAFIIQGLDRTQQKLAQQVSFRLAEVWLRSNYKSFAEK 533

||||.||||||:|.|:|||||||....||:|::..|:.|:.:||..:.:.

BtTrehalase 486 QQWDQPNAWPPVQVFLIQGLDRTGVPQAQEVAYSFAQNWVHTNYLGYKKA 535

ApTrehalase 534 SMMFEKYDVLASGETGGGGEYTPQTGFGWTNGVVFEFLNRWGDTL-SNGI 582

..|||||.:..:|||||||||.||||||||||||||.|:|:.|.| :|..

TrehalaseBemi536 GFMFEKYHIRLAGETGGGGEYEPQTGFGWTNGVVFEMLDRYSDRLTANYK 585

ApTrehalase 583 NDLRRHG 589

.:::

BtTrehalase 586 FEVQ--- 589
